# Detachment of a prosthetic valve due to infective endocarditis caused by *Streptococcus pneumoniae*

**DOI:** 10.1002/ccr3.2749

**Published:** 2020-07-10

**Authors:** Saori Nakamura, Naoji Hanayama, Hiroshi Matushita, Kenjiro Sakaki

**Affiliations:** ^1^ Department of Cardiothoracic Surgery Kanto Rosai Hospital Kanagawa Japan; ^2^ Department of Cardiology Tokyo Metropolitan Tama Medical Center Tokyo Japan

**Keywords:** aortic valve replacement, cardiac surgery, prosthetic valve endocarditis, *Streptococcus pneumoniae* infection

## Abstract

The incidence of infective endocarditis (IE) due to *S pneumoniae* has decreased, thanks to antibiotics. However, when it does occur, it can be lethal. The present case provides a reminder of the potential lethality of this postoperative infection.

## Graphical abstract

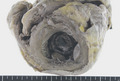

## 1 INTRODUCTION

We report a case of a 69‐year‐old female patient with *Streptococcus pneumoniae*‐positive infective endocarditis following aortic valve replacement. Her symptoms gradually improved. However, she died due to a prosthetic valve deviation caused by the infection. Physicians should be alert to the possibility that it may follow a lethal course.

The incidence of infective endocarditis caused by pneumococcal bacteria has been on the decline due to the judicious use of penicillin therapy.^1^ However, infective endocarditis caused by pneumococci in an aortic valve tends to have an acute course.^2^, ^3^ We reported herein a case of prosthetic aortic valve detachment due to pneumococcal infective endocarditis.

## 2 CASE PRESENTATION

A 69‐year‐old female patient presented with fever, headache, nausea, and vomiting of several days’ duration. She had a past medical history of aortic valve replacement with a mechanical valve due to aortic regurgitation 4 years prior to admission. When she was admitted to the hospital, she presented with hypotension, with a systolic blood pressure as low as 80 mm Hg. Her blood pressure increased after fluid replacement was initiated due to suspected intravascular dehydration based on the finding of collapsed inferior vena cava. Heart murmurs and abdominal tenderness were denied, and the physical findings revealed no clue as to the cause of her fever. Blood tests denied any increase in total bilirubin (0.4 mg/dL) but confirmed an increased in hepatobiliary enzymes, including AST (153 IU/L), ALT (128 IU/L), and ALP (500 IU/L).

**Figure 1:**
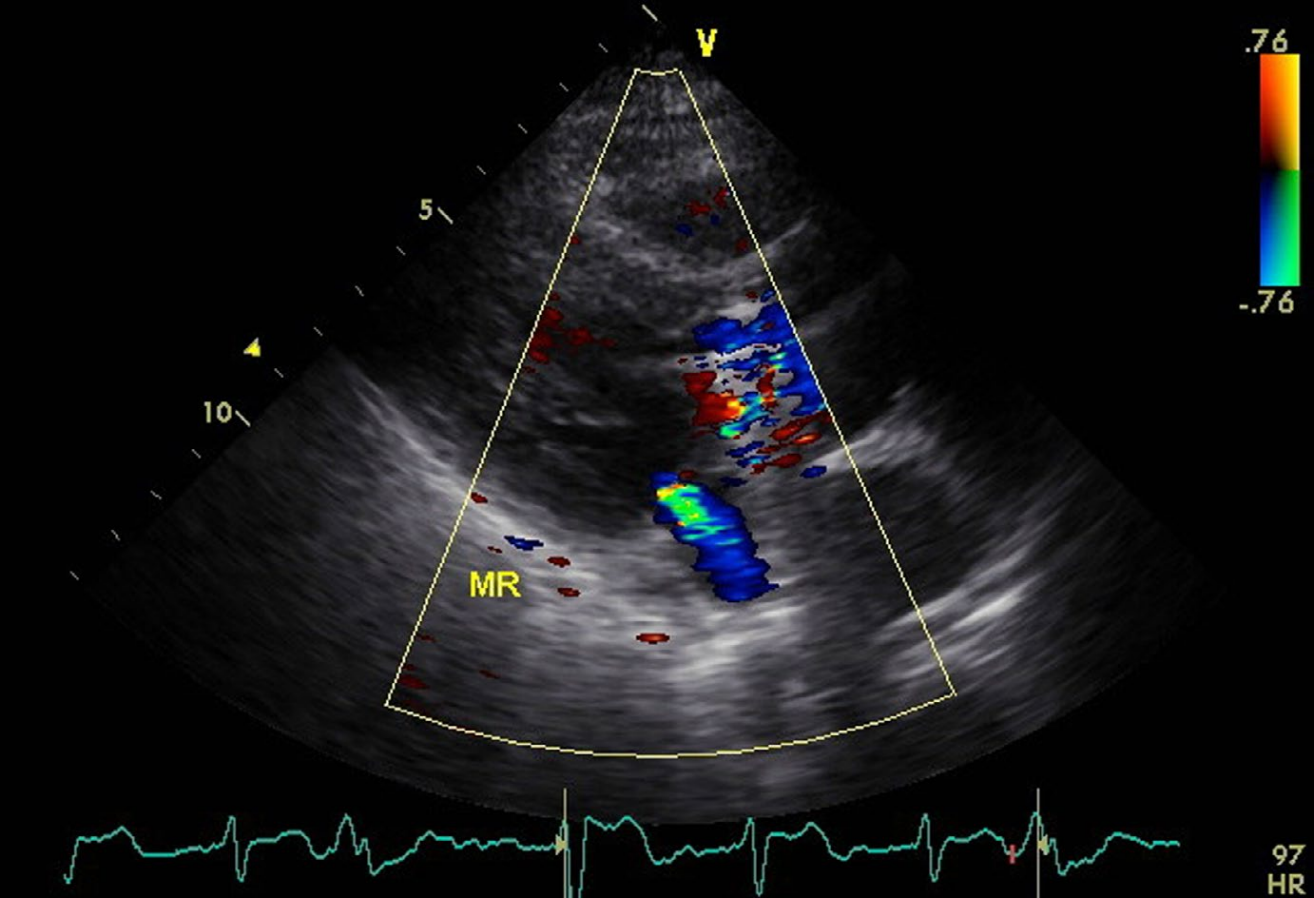
Echo image on hospital day 2. No vegetation was detected, and no aortic regurgitation was observed

Furthermore, computed tomography (CT) suggested cholecystitis due to gallbladder expansion. Even though her previous aortic valve surgery led us to consider the possibility of IE, transthoracic echocardiography (TTE) denied vegetation. (Figure 1) Transesophageal transthoracic echocardiography (TEE) was not performed because the patient was unable to give her consent due to severe nausea.

Based on the elevated hepatobiliary enzymes and CT results, treatment for cholecystitis was started with cefmetazole.

The hepatobiliary enzymes peaked on day 2 of admission. On day 3, gram‐positive cocci were detected in a blood culture. The presence of enterococci from cholecystitis was suspected, and vancomycin administration was started. Thereafter, the patient's symptoms gradually improved. On day 5, penicillin‐susceptible *Streptococcus pneumoniae* were detected in a blood culture, and vancomycin was switched to penicillin. At this point, a serial blood culture was again performed, but the results were later confirmed to be negative.

**Figure 2:**
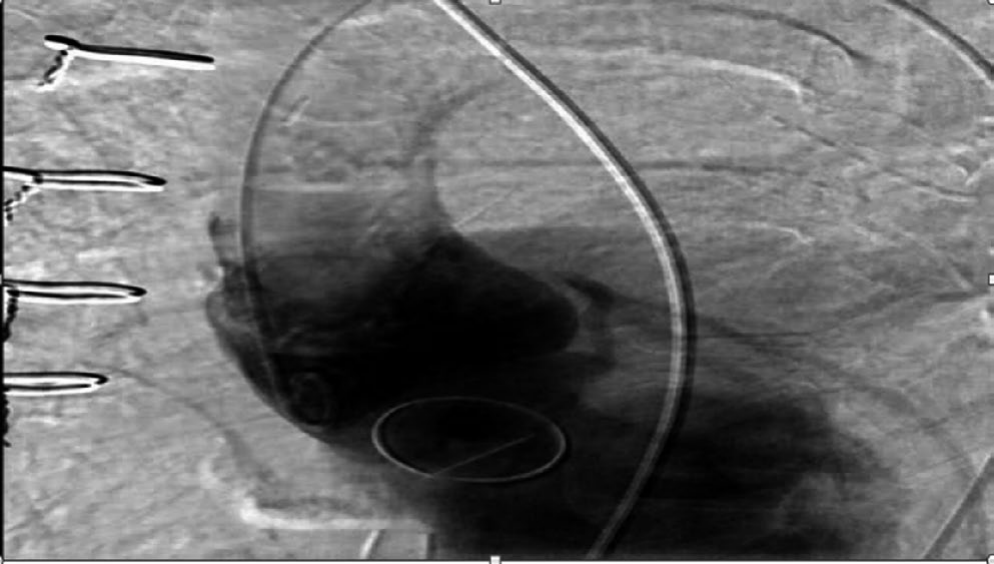
Aortic angiography after cardiac arrest

The patient experienced a convulsion on day 6, and endotracheal intubation was done due to her deteriorating consciousness status, reduced respiratory effort, and persistent hypotension. Pulseless electrical activity (PEA) was noted during the intubation, and resuscitation was started immediately. When resuscitation proved unsuccessful, intra‐aortic balloon pump (IABP) and percutaneous cardiopulmonary support (PCPS) were implemented to stabilize the patient's condition. Contrast CT revealed no identifiable causes of the cardiac arrest, including pulmonary embolism and acute aortic dissection. Moreover, a coronary angiography showed no lesions. At the time of the aortography, no aortic dissection was observed, and the deviation of the valve was not noticed at this point. (Figure 2).

The patient was stabilized in the intensive care unit and closely monitored. The PCPS flow was unable to be maintained, and the patient became bradycardic despite aggressive fluid resuscitation, including a blood transfusion. The arterial blood gas showed severe metabolic acidosis, with pH 6.83 and lactate 10.9 mmol/L. Unfortunately, the patient died despite our strenuous efforts at resuscitation.

**Figure 3:**
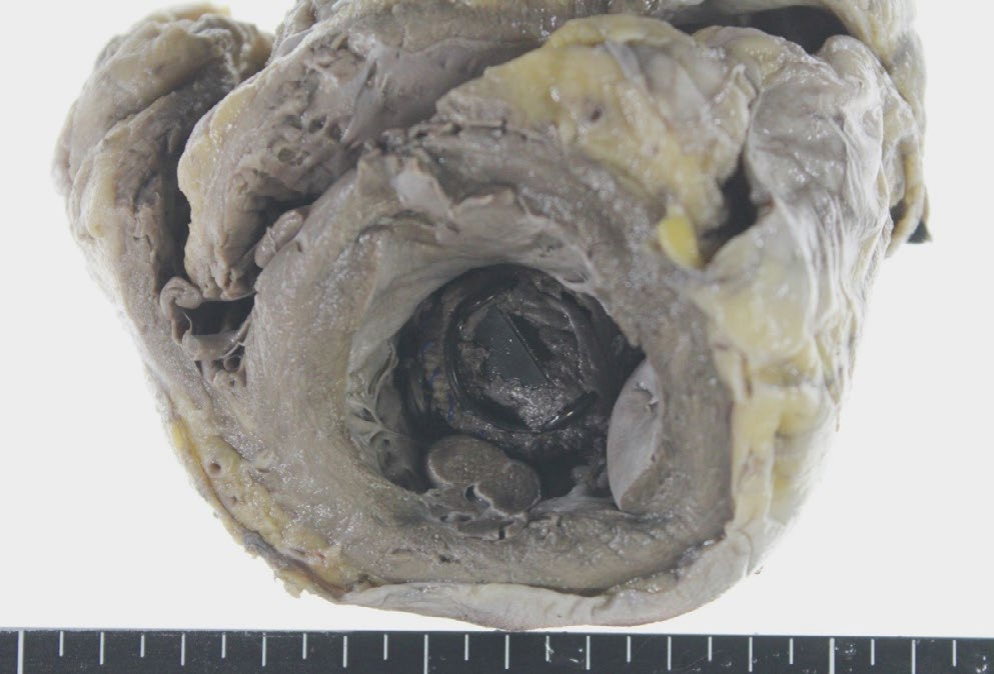
Cross‐section of left ventricle. Detachment of the artificial aortic valve can be seen

An autopsy showed deviation of the artificial aortic valve toward the left ventricle. (Figure 3) Although this change was thought to be associated with cardiopulmonary resuscitation, microscopy revealed the formation of micro‐abscesses with marked neutrophil infiltration from the epicardium to the myocardium and the presence of fibrous tissue at the valve margins which had altered the shape of the valve. Debris consisting of collections of neutrophils was also attached to the prosthetic valve, but the presence of a bacterial mass was unable to be confirmed even with Gram staining.

These results indicated that the cause of death was aortic valve detachment due to the infiltration of inflammatory cells associated with infective endocarditis.

## 3 DISCUSSION

The present patient's condition initially seemed to be stable but rapidly deteriorated. Based on the clinical findings and pathological autopsy results, the inflammation of the aortic valve was found to be caused by infective endocarditis due to *Streptococcus pneumoniae,* with the inflamed tissue becoming weakened and leading to the dehiscence of the prosthetic valve. The so‐called Austrian syndrome, a combination of meningitis, pneumonia, and infective endocarditis due to *Streptococcus pneumoniae* infection, was often observed in the prepenicillin era.^4^ As penicillin became widely available, the incidence of infective endocarditis due to *Streptococcus pneumoniae* decreased; currently, about 1.3% of invasive pneumococcal infection cases progress to infective endocarditis, and <3% of infective endocarditis cases are due to *Streptococcus pneumoniae*.^1^, ^2^

However, one of the potential complications of infective endocarditis caused by pneumococci is inflammation of the aorta, which can have a lethal course, especially in patients with an aortic valve replacement.^2^, ^3^, ^5^ In addition, it is known that the incidence of infective endocarditis increases significantly in patients following valve replacement^6^; hence, careful monitoring of these patients is crucial.

Cardiac ultrasonography aids the diagnosis of infective endocarditis and is included in the Duke classification. In patients with a cardiac prosthetic implant or devices such as an artificial valve or pacemaker, transesophageal echocardiography (TEE) can be used for detailed observation. The sensitivity and specificity of TEE range from 57% to 86% and 63% to 88%, respectively.^7^, ^8^ However, in the present case, the TEE was unable to be performed because the patient was incapable of giving her consent due to severe nausea.

In recent years, CT and 18 F‐fluorodeoxyglucose (18 F‐FDG) PET/CT are reportedly useful for visualizing abscesses of the valve annulus.^9^ However, PET/CT is not always available in municipal general hospitals.

There are two main points to consider in this case. The first is the timing of the transesophageal echocardiography. An emergency transesophageal echocardiography was not performed due to the patient's persistent nausea and the rarity of PSSP as a cause of endocarditis. If it had been done during the patient's hospitalization, it might have detected the abscess around the valve. Second, the valve deviation was not observed at the time of the aortography. Valve dehiscence should be considered as a cause of sudden cardiac arrest, and aortic dissection and coronary occlusion should be ruled out. Infective endocarditis due to *S pneumoniae* is decreasing in incidence but should not be underestimated. In particular, the possibility of IE should be considered in patients with a history of valve replacement. Furthermore, it is important to examine for valve dehiscence carefully in cases of sudden cardiac arrest in patients with a prosthetic valve.

## 4 CONCLUSION

The incidence of IE due to *Streptococcus pneumoniae* after valve replacement surgery has largely decreased in recent years, thanks to the timely use of antibiotics. We reported herein an unusual case of a 69‐year‐old female patient with infective endocarditis due to *Streptococcus pneumoniae* following aortic valve replacement. The pathology report revealed that the cause of the prosthetic valve detachment was a *S pneumoniae* infection. The present case provides a reminder of the potential lethality of postoperative infective endocarditis due to *Streptococcus pneumoniae*.

## CONFLICT OF INTEREST

None declared.

## AUTHOR CONTRIBUTIONS

SN: drafted the manuscript; HM and KS: provided clinical support; NH: reviewed the manuscript; all the authors approved the submission of the manuscript and agree to be accountable for all aspects of the work by ensuring that questions related to the accuracy or integrity of any part of the work are appropriately investigated and resolved.
